# Metastasis of Ovarian Cancer to the Duodenum: A Case Report

**DOI:** 10.70352/scrj.cr.25-0581

**Published:** 2025-11-07

**Authors:** Hiromitsu Maehira, Haruki Mori, Riho Shiroyama, Nobuhito Nitta, Tsukuru Amano, Shunichiro Tsuji, Takeru Maekawa, Takeshi Sonoda, Sachiko Kaida, Toru Miyake, Katsushi Takebayashi, Masatsugu Kojima, Reiko Otake, Soichiro Tani, Masaji Tani

**Affiliations:** 1 Department of Surgery, Shiga University of Medical Science, Otsu, Shiga, Japan; 2 Department of Obstetrics and Gynecology, Shiga University of Medical Science, Otsu, Shiga, Japan

**Keywords:** duodenum metastasis, ovarian cancer, curative resection

## Abstract

**INTRODUCTION:**

Ovarian cancer remains a leading cause of cancer-related mortality. Although the peritoneum and abdominal lymph nodes are the most common sites of metastasis, duodenal metastasis is extremely rare. We report a rare case of duodenal metastasis from ovarian clear cell carcinoma (OCCC) that initially presented with gastrointestinal bleeding and was successfully treated with curative resection.

**CASE PRESENTATION:**

A 52-year-old woman with a history of OCCC (International Federation of Gynecology and Obstetrics stage IIA) underwent total hysterectomy with bilateral salpingo-oophorectomy and partial omentectomy, followed by adjuvant platinum-based chemotherapy. During follow-up, routine blood tests revealed marked anemia. Contrast-enhanced CT and MRI demonstrated a 30-mm tumor in the second portion of the duodenum with pancreatic invasion. Upper gastrointestinal endoscopy revealed a type II ulcerative tumor with peripheral bleeding, and biopsy confirmed clear cell carcinoma. Given the anemia caused by tumor bleeding, pancreaticoduodenectomy was performed with curative intent. Histopathological findings were consistent with metastatic OCCC. The patient has remained recurrence-free for 30 months postoperatively.

**CONCLUSIONS:**

We present a rare case of platinum-resistant OCCC with duodenal metastasis that was successfully managed with pancreaticoduodenectomy, resulting in long-term survival. Although the role of surgical resection in such cases remains uncertain, this case suggests that curative surgery may be beneficial in selected patients.

## Abbreviations

CA125
: cancer antigen 125
CDX2
: caudal type homeobox 2
CK7
: cytokeratin 7
CK20
: cytokeratin 20
OCCC
: ovarian clear cell carcinoma

## INTRODUCTION

Among gynecologic malignancies, ovarian cancer remains the leading cause of cancer-related mortality.^1)^ Although the standard treatment typically involves cytoreductive surgery followed by combination chemotherapy with carboplatin and paclitaxel, advanced-stage disease is often associated with poor prognosis and a high recurrence rate.^2)^ In particular, the clear cell subtype of ovarian carcinoma exhibits marked resistance to platinum-based chemotherapy, contributing to unfavorable clinical outcomes.^3)^

Although the peritoneum and abdominal lymph nodes are the most frequent sites of metastasis,^4)^ duodenal metastasis is exceedingly rare. We describe a rare case of duodenal metastasis from ovarian cancer that initially presented with gastrointestinal bleeding and was successfully treated with curative resection, resulting in prolonged survival.

## CASE PRESENTATION

A 52-year-old woman with a medical history of hypertension was previously diagnosed with International Federation of Gynecology and Obstetrics Stage IIA OCCC. Three years earlier, she underwent total hysterectomy with bilateral salpingo-oophorectomy and partial omentectomy. Postoperatively, the patient received 6 cycles of combination chemotherapy with paclitaxel and carboplatin. Nine months after the initial surgery, liver metastasis was detected, and laparoscopic partial hepatectomy was performed. Following hepatectomy, the patient was treated with doxorubicin and bevacizumab; however, this regimen was discontinued due to a subarachnoid hemorrhage, and the patient was placed under regular monitoring.

**Fig. 1:**
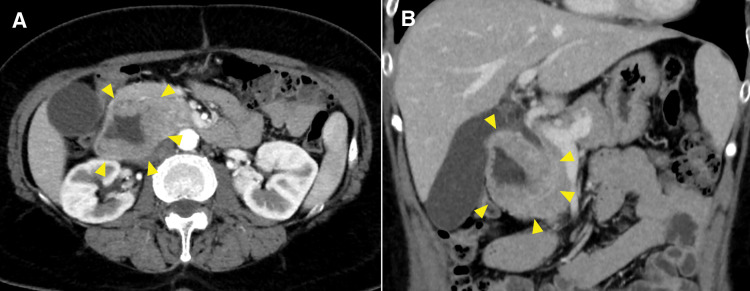
Contrast-enhanced CT findings. Contrast-enhanced CT shows a tumor in the second portion of the duodenum measuring 30 mm in diameter, with persistent enhancement and invasion of the pancreas (yellow arrowheads). (**A**) Axial view, arterial phase; (**B**) coronal view, portal phase.

**Fig. 2:**
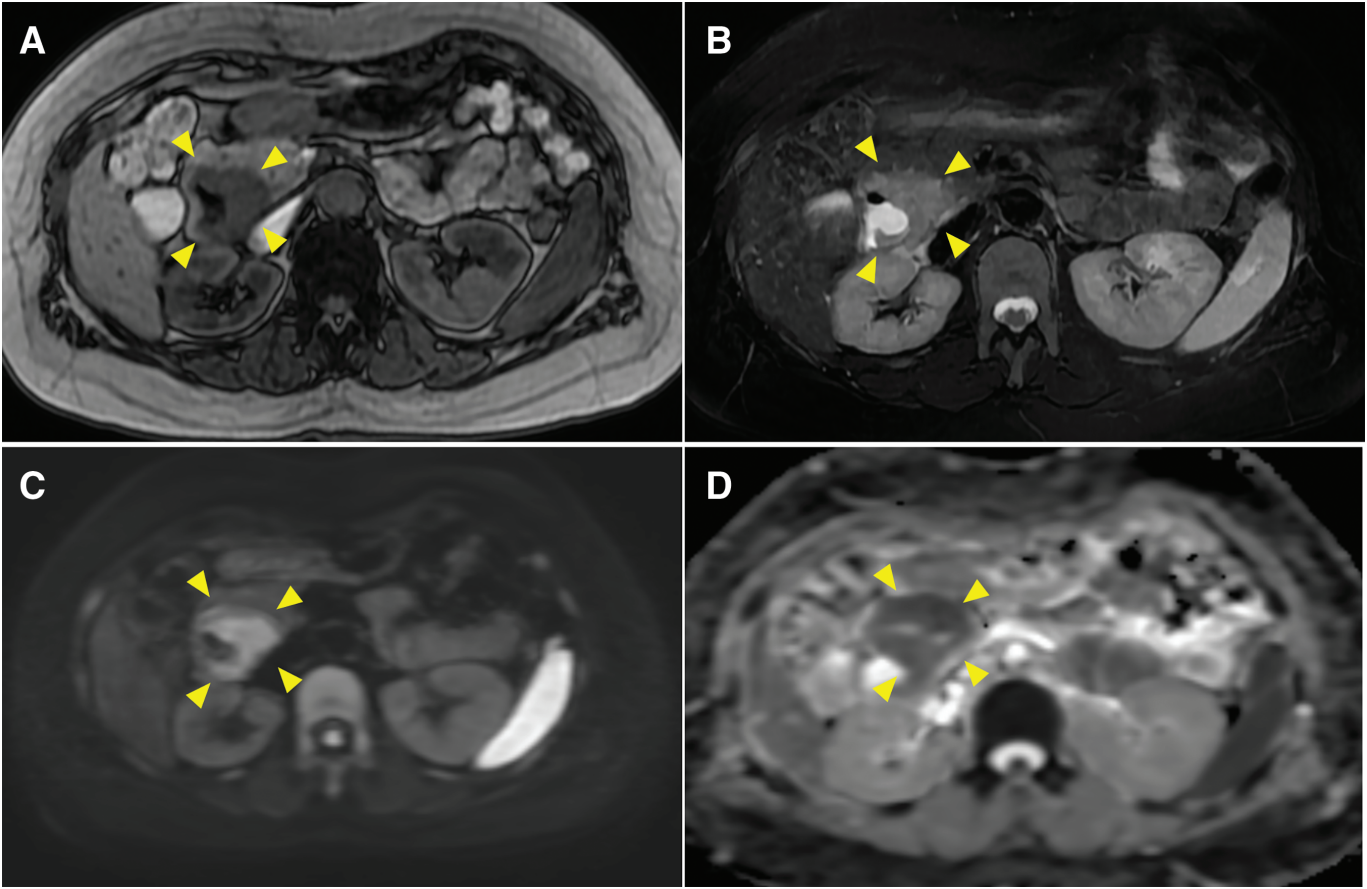
MRI findings. MRI shows a hypointense signal on T1-weighted images, a mildly hyperintense signal on T2-weighted images, high signal intensity on diffusion-weighted imaging, and low signal intensity on apparent diffusion coefficient map in the 2nd portion of the duodenum (yellow arrowheads). (**A**) Axial view, T1-weighted image; (**B**) axial view, T2 weighted image; (**C**) axial view, diffusion-weighted image; (**D**) axial view, apparent diffusion coefficient maps.

**Fig. 3:**
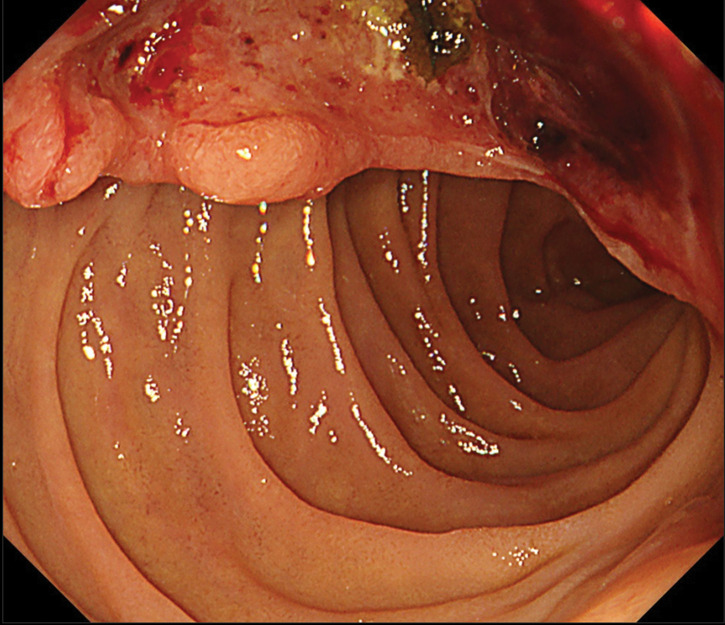
Upper gastrointestinal endoscopy findings. Upper gastrointestinal endoscopy shows a type II tumor with peripheral bleeding.

**Fig. 4:**
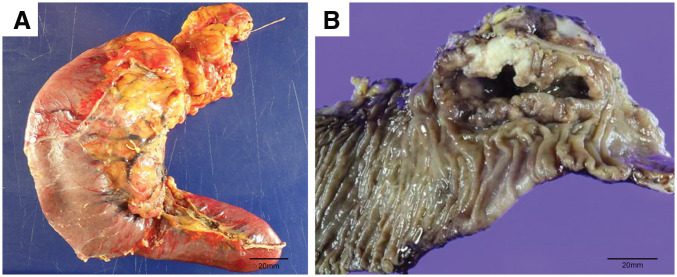
Macroscopic findings of the resected specimen. (**A**) Gross specimen prior to formalin fixation. The serosal surface of the duodenum appears normal. (**B**) Formalin-fixed specimen. The tumor size was 30 × 80 mm.

The patient had been followed up every 4 months, but 30 months after the initial surgery, routine blood tests revealed a sudden drop in hemoglobin level from the usual 13.0 g/dL to 4.8 g/dL. Regarding the tumor marker, the serum CA125 level was within the normal limit. Contrast-enhanced CT revealed a solitary tumor in the second portion of the duodenum, measuring 30 mm in diameter, with persistent pancreatic enhancement and invasion (**Fig. 1**). Although no bile duct dilatation was observed, the tumor compressed the common bile duct. MRI revealed a hypointense signal on T1-weighted images, a mildly hyperintense signal on T2-weighted images, a hyperintense signal on diffusion-weighted images, and a hypointense signal on apparent diffusion coefficient maps in the 2nd portion of the duodenum (**Fig. 2**). Upper gastrointestinal endoscopy revealed a type II tumor with peripheral bleeding (**Fig. 3**), and biopsy confirmed clear cell carcinoma. Based on these findings, the lesion was diagnosed as a duodenal metastasis of OCCC. When the hemoglobin level dropped to 4.8 g/dL, blood transfusion and iron supplementation were administered, resulting in an improvement to 9.3 g/dL. However, 1 month later, the hemoglobin level decreased again to 7.3 g/dL, necessitating another transfusion. Although the hemoglobin level subsequently increased to 10.3 g/dL, it had fallen to 7.9 g/dL just before surgery. Given the presence of anemia caused by tumor bleeding, pancreaticoduodenectomy was performed 2 months after the duodenal lesion had been detected on CT (**Fig. 4**). The operative time was 297 minutes, and the estimated blood loss was 226 mL. No postoperative complications were observed.

**Fig. 5:**
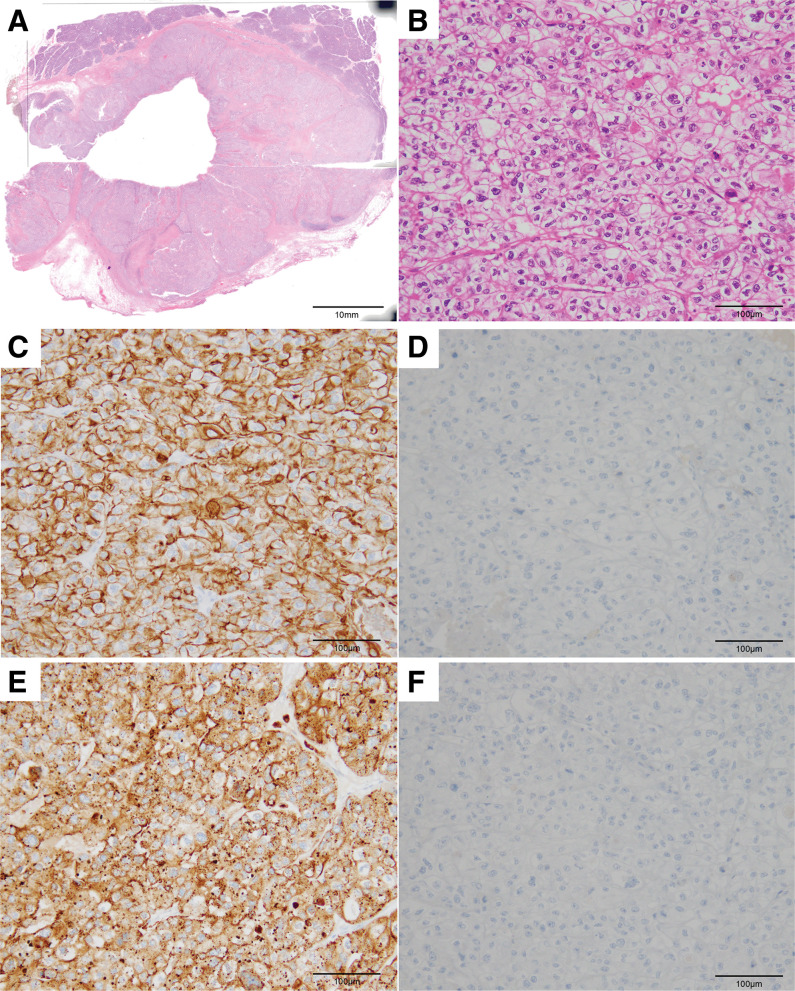
Histopathological and immunohistochemical findings. (**A**) Histopathological findings show a duodenum tumor. The serosal surface of the duodenum appears normal. Hematoxylin and eosin staining, original magnification. (**B**) The tumor is composed of clear cells with enlarged, variably sized hyperchromatic nuclei. Hematoxylin and eosin staining, original magnification ×200. (**C**) Immunohistochemical findings show that the tumor cells are positive for the expression of CK7; (**D**) negative for the expression of CK20; (**E**) positive for the expression of Napsin A; and (**F**) negative for the expression of CDX2. Original magnification ×200. CDX2, caudal type homeobox 2; CK7, cytokeratin 7; CK20, cytokeratin 20

Histopathological examination revealed that the tumor was composed of clear cells with enlarged, variably sized hyperchromatic nuclei (**Fig. 5B**). The duodenal serosal surface was normal (**Fig. 5A**). Immunohistochemical staining revealed CK7 positivity, CK20 negativity, Napsin A positivity, and CDX2 negativity, consistent with metastatic OCCC in the duodenum (**Fig. 5C**–**5F**).

Postoperatively, the patient received adjuvant chemotherapy with nogitecan and bevacizumab, which was discontinued after 2 cycles due to bone marrow suppression. She has since been under observation, with no signs of recurrence 30 months after pancreaticoduodenectomy.

## DISCUSSION

We report a rare case of OCCC with duodenal metastasis. Metastasis of ovarian cancer to the upper gastrointestinal tract is extremely rare, and to date, no large-scale study has reported its exact incidence. Metastatic lesions in the stomach from all malignant tumors are reported in approximately 1% of autopsy cases, most commonly originating from the breast, melanoma, and lung.^5,6)^ Therefore, duodenal metastasis from ovarian cancer appears to be an exceptionally uncommon event, regardless of histological subtype. In our case, complete surgical resection of the metastatic lesion was achieved, resulting in long-term survival. This case suggests that curative resection may be a viable option, even for rare metastatic sites.

OCCC is a relatively rare histological subtype of epithelial ovarian cancer.^7,8)^ In advanced stages, OCCC exhibits greater resistance to platinum-based chemotherapy than other subtypes, with a reported median survival time of 26.8 months and a 5-year survival rate of only 30.1%, indicating a generally poor prognosis.^9)^ In the present case, although adjuvant chemotherapy with carboplatin and paclitaxel was administered after the initial surgery, hepatic recurrence occurred within 9 months, suggesting platinum resistance and a poor anticipated outcome.

The most common metastatic sites of ovarian cancer are the peritoneum and abdominal lymph nodes, whereas metastasis to the upper gastrointestinal tract is rare.^4)^ Among such rare presentations, duodenal metastasis is extremely uncommon, with only a few cases reported to date (**Table 1**).^10–13)^ The therapeutic strategies for duodenal metastasis varied among the cases. Two patients including our case underwent pancreaticoduodenectomy, while others received palliative therapy or best supportive care. When ovarian cancer metastasizes to the upper gastrointestinal tract, the symptoms are often nonspecific, including abdominal pain, vomiting, anorexia, gastrointestinal bleeding, or anemia, which may delay diagnosis in many cases.^14)^ Furthermore, serum CA125 level had been reported to be elevated in approximately 75% of patients with high-grade serous ovarian carcinoma.^15)^ However, its diagnostic and monitoring value is considered less significant in OCCC. In the present case, the CA125 level remained within the normal range throughout the clinical course, suggesting that this marker was not considered useful for postoperative follow-up in this patient. Therefore, in patients with ovarian cancer who present with abdominal symptoms, the possibility of gastrointestinal metastasis should be considered during the differential diagnosis.

**Table 1:** Literature review of duodenal metastasis from ovarian cancer

	Author	Tumor status at the time of duodenal metastasis detection	Detection timing of duodenal metastasis after primary resection	Treatment for duodenal metastasis	Histological type
1	Barbeiro et al.^10)^	Ovarian cancer, Peritoneal metastasis	NA	Best supportive care	ND
2	Takasaki et al.^11)^	Post-resection of ovarian cancer, Post-resection of paraaortic lymph node metastasis	264 months	Pancreaticoduodenectomy	Adenocarcinoma
3	Kolli et al.^12)^	Ovarian cancer, Brain metastasis	NA	Best supportive care	Serous adenocarcinoma
4	Puvvada et al.^13)^	Post-resection of ovarian cancer, Post-resection of liver metastasis	48 months	Chemotherapy, palliative radiotherapy	Serous adenocarcinoma
5	Our case	Post-resection of ovarian cancer, Post-resection of liver metastasis	30 months	Pancreaticoduodenectomy	Clear cell carcinoma

NA, not available; ND, not described

The precise mechanism of duodenal metastasis in ovarian cancer remains unclear. In our case, the liver metastasis was considered hematogenous because it was located within the liver parenchyma. In duodenal metastasis, no gross or histopathological abnormalities were observed on the serosal duodenal surface; therefore, peritoneal dissemination was not suspected. Lymphatic or hematogenous spread is considered more likely; however, the exact pathway remains uncertain. Further case reports and investigations are warranted to elucidate the underlying mechanisms.

Surgical resection of metastatic lesions in recurrent ovarian cancer with platinum sensitivity has been reported to improve the prognosis.^16)^ However, the efficacy of surgical resection in platinum-resistant recurrent ovarian cancer has not been established, and no large-scale randomized controlled trials have been conducted in this setting. In the present case, duodenal metastasis was detected 2 years after hepatic metastasectomy. As the metastatic lesion invaded the pancreas, pancreaticoduodenectomy, a highly invasive procedure, was required to achieve complete resection. Although radiotherapy was considered as a hemostatic treatment for the duodenal bleeding, surgical resection was ultimately chosen because the anemia was progressing rapidly, the reported hemostatic control rate of radiotherapy was approximately 80%,^17,18)^ and the patient was young and in good general condition, making surgery a feasible option. For elderly patients or those with poor general condition in whom surgery is not indicated, palliative radiotherapy or best supportive care may be considered as alternative therapeutic options. Complete resection and long-term survival were achieved in this patient. While curative resection for duodenal metastasis may require extensive surgery, selected cases, even those with platinum-resistant disease, could still derive survival benefits from such intervention. As this is an extremely rare condition, further evaluation will require the accumulation of cases through multicenter retrospective cohort studies.

## CONCLUSIONS

We encountered a rare case of platinum-resistant OCCC with duodenal metastasis that was discovered because of progressive anemia and successfully managed with pancreaticoduodenectomy, resulting in long-term survival. The route of ovarian cancer metastasis to the duodenum remains unclear, and the clinical significance of surgical resection for such metastases is uncertain due to the limited number of reported cases. Further accumulation of cases and investigations is needed to better define metastatic patterns and clarify the role of surgical intervention in similar scenarios.

## DECLARATIONS

### Availability of data and materials

Data sharing is not applicable, as no datasets were generated or analyzed in this study.
